# Refinement of analgesia following thoracotomy and experimental myocardial infarction using the Mouse Grimace Scale

**DOI:** 10.1113/expphysiol.2014.083139

**Published:** 2015-01-14

**Authors:** Kiterie M. E. Faller, Debra J. McAndrew, Jurgen E. Schneider, Craig A. Lygate

**Affiliations:** ^1^ Division of Cardiovascular MedicineRadcliffe Department of MedicineBritish Heart Foundation Centre of Research Excellence and Wellcome Trust Centre for Human GeneticsUniversity of OxfordUK; ^2^ School of Veterinary MedicineCollege of MedicalVeterinary and Life SciencesUniversity of GlasgowUK

## Abstract

**New Findings:**

- **What is the central question of this study?**
  There is an ethical imperative to optimize analgesia protocols for laboratory animals, but this is impeded by our inability to recognize pain reliably. We examined whether the Mouse Grimace Scale (MGS) provides benefits over a standard welfare scoring system for identifying a low level of pain in the frequently used murine surgical model of myocardial infarction.
- **What is the main finding and its importance?**
  Low‐level pain, responsive to analgesia, was detected by MGS but not standard methods. In this model, most of the pain is attributable to the thoracotomy, excepted in mice with very large infarcts. This approach represents a model for assessing postsurgical analgesia in rodents.

The Mouse Grimace Scale (MGS) was developed for assessing pain severity, but the general applicability to complex postsurgical pain has not been established. We sought to determine whether the MGS provides benefits over and above a standard welfare scoring system for identifying pain in mice following experimental myocardial infarction. Female C57BL/6J mice (*n* = 60), anaesthetized with isoflurane, were subjected to thoracotomy with ligation of a coronary artery or sham procedure. A single s.c. dose of buprenorphine (1.1 mg kg^−1^) was given at the time of surgery and pain assessed at 24 h by MGS and a procedure‐specific welfare scoring system. In some animals, a second dose of 0.6 mg kg^−1^ buprenorphine was given and pain assessment repeated after 30 min. The MGS was scored from multiple photographs by two independent blinded observers with good correlation (*r* = 0.98). Using the average MGS score of both observers, we identified a subset of mice with low scores that were not considered to be in pain by the welfare scoring system or by single observer MGS. These mice showed a significant improvement with additional analgesia, suggesting that this low‐level pain is real. Pain attributable to the myocardial injury, as opposed to thoracotomy, persisted at 24 h only in mice with large infarcts >40%. In conclusion, the use of a multi‐observer, *post hoc* version of the MGS is a sensitive tool to assess the efficacy of postsurgical analgesic protocols. Following surgical induction of myocardial infarction, we identified a significant proportion of mice that were in low‐level pain at 24 h that were not identified by other assessment methods.

## Introduction

Effective pain management in laboratory rodents is crucial not only for ethical and legal considerations but also in order to achieve high‐quality science free from the confounding pathophysiological consequences of pain (Carbone, 2011). The development of the ARRIVE guidelines (Kilkenny *et al*. 2010), the refinement of pre‐existing guidelines (e.g. *Guide for the Care and Use of Laboratory Animals*, 8th edn, published by the US National Academy of Sciences, Institute for Laboratory Animal Research, 2011; Carbone, 2012) and the implementation of new directives (e.g. European Directive 2010/63/EU on the protection of animals used for scientific purposes) have all further emphasized the importance of the ‘3Rs’ (replacement, reduction and refinement) in biomedical research.

Surgical ligation of a major coronary artery in the mouse is a widely used model of experimental myocardial infarction (MI) and consequent congestive heart failure. Despite the fact that thoracotomy is considered one of the most painful surgical procedures in humans (Gerner, 2008) and that pain from myocardial infarction may be ‘severe and persistent’ (Alderman, 1974), the extent to which postoperative analgesia is provided for mice is highly variable and often goes unreported in the published literature (Patten *et al*. 1998; Salto‐Tellez *et al*. 2004; Karas, 2006). Of particular concern is the ability of mice, as a prey species, to adapt and mask pain‐related behaviours (Roughan & Flecknell, 2001), which is likely to result in an underestimation of pain following this type of procedure. A major problem for treating pain effectively in laboratory animals is therefore the ability to recognize pain reliably. The current ‘gold‐standard’ approach is to combine assessment of behavioural and physiological parameters in specifically designed score sheets; however, these are often considered time consuming and highly subjective by animal care staff (Hawkins, 2002). Recently, a new method to evaluate animal pain based on facial expression, the Mouse Grimace Scale (MGS), was described and validated in several experimental models of inflicted pain. It was considered best suited to assess pain which lasted up to 48 h (Matsumiya *et al*. 2012) and compared favourably with extensive and complex manual and automatic behavioural analysis (Leach *et al*. 2012). However, the MGS has never been compared with a more standard pain‐scoring scheme, such as those most frequently used in animal research facilities. Furthermore, its utility to assess complex surgical pain of longer duration needs to be assessed.

Therefore, the aims of this study were as follows: (i) to validate the Mouse Grimace Scale in evaluating pain 24 h following MI in mice; (ii) to assess how the MGS compares with a more traditional welfare score sheet specifically designed for MI; and (iii) to assess the efficacy of buprenorphine in treating pain induced by the MI procedure in order to optimize postsurgical analgesic protocols.

## Methods

### Ethical approval

All experiments were approved by the institutional ethical review committee of the University of Oxford and conform with the UK Home Office Guidance on the Operation of the Animals (Scientific Procedures) Act, 1986 incorporating European Directive 2010/63/EU (licence number 30/2754). No mice underwent surgery for the purposes of this study. All mice were part of an on‐going separate study of chronic heart failure, which required them to be kept for 8 weeks following myocardial infarction. Any mouse showing signs of distress, particularly dyspnoea, weight loss or decreased activity, was killed immediately as a humane end‐point by cervical dislocation. As determined by the needs of the separate study, animals were killed at the end of the 8 week experiment by exsanguination under an overdose of inhaled anaesthetic agent (5% isoflurane). No untreated control animals were used, i.e. all mice received our standard practice of at least one dose of long‐acting perisurgical analgesia.

### Animal husbandry

Mice were either C57BL/6J obtained from Harlan UK or were transgenic mice bred in‐house overexpressing creatine transporter in the heart with a genetic background congenic to C57BL/6J (as described by Wallis *et al*. 2005). Transgene expression in this model is restricted to cardiomyocytes and is therefore highly unlikely to influence pain perception. Mice were socially housed (two to five animals per cage) in a specific pathogen‐free environment, with controlled humidity and temperature (20–22 °C) and a 12 h–12 h light–dark cycle. All mice were adult females with body weight at time of surgery 22 ± 2 g (i.e. approximately 3–4 months old).

### Mouse groups

Four groups of mice were studied. There were two surgical groups; one group was subjected to thoracotomy with ligation of a coronary artery to induce myocardial infarction (MI group), and a second group received thoracotomy only (sham group). Two non‐surgical groups were used as additional controls; the first received identical general anaesthesia (GA) using isoflurane as part of a magnetic resonance imaging (MRI) examination but did not undergo any surgical procedures (GA only group), and the last group consisted in mice not under any protocol (stock control group).

### Myocardial infarction surgery

All surgeries were performed in the morning. Permanent coronary artery ligation was performed by the same person, as previously described in detail (Lygate, 2006; Lygate & Neubauer, 2006). In brief, general anaesthesia was induced with 4% isoflurane, then maintained at 2% in 100% O_2_. Mice received 0.024 mg buprenorphine s.c. (i.e. average dose, 1.1 mg kg^−1^; Vetergesic, Alstoe Animal Health, UK), intubated and ventilated with a tidal volume of 250 μl and respiratory rate 150 breaths min^−1^ (Hugo‐Sachs MiniVent type 845; Harvard Apparatus Ltd., Kent, UK). A left thoracotomy was performed in the fourth intercostal space, the pericardium removed, and an intramyocardial ligature placed 1–2 mm below the atrioventricular groove using a 6–0 polyethylene suture (Ethicon, Johnson & Johnson Medical Ltd., Wokingham, UK). Another group of sham mice underwent an identical protocol without ligation of the coronary artery. Mice were provided with supplemental heat overnight, and pain scoring was performed the following morning, i.e. 24 h after surgery and initial buprenorphine analgesia.

### Traditional behavioural and clinical pain scoring

All mice were scored using a traditional welfare scoring system (Table 1). This system has been created by combining criteria frequently used to assess pain or discomfort in laboratory rodents (Morton, 1999; Hawkins, 2002; Wolfensohn & Lloyd, 2003). Only criteria considered relevant for the model assessed were used, and the scoring system was tested and optimized prior to the start of this study. Assessment was performed in the following three steps: by observing mice from a distance, at cage opening and at handling. Each criterion was marked as absent (0), mildly present or doubtful (1) or present (2). The importance of breathing and mucous membrane colour was emphasized by doubling the value for these criteria due to the nature of the surgery performed. All mice received a mark out of 50.

**Table 1:** **Traditional welfare scoring system based on behavioural, clinical and procedure‐specific criteria**

Observation from a distance (for 5 min)
(1) Inactive
(2) Isolated
(3) Hunched posture
(4) Huddled
(5) Restless
(6) Reluctance to move
(7) Laboured gait/tip‐toe walking
(8) Abnormal interaction with congeners (other than isolation; specify)
(9) Excessive attention to surgical site
Observation following cage lid opening
Reaction at cage opening:
(10) Little, no response (not inquisitive, nor alert)
(11) Hyperactive
(12) Starey coat/piloerection
(13) Not grooming
(14) Twitching/tremors
(15) Type of breathing: normal (N), laboured (L; slow, involving the diaphragm but with closed mouth), open‐mouth breathing (O), noisy breathing (crackles; C).
Observation following handling
Reaction to handling:
(16) Reacts violently (attempt to bite, irritation)
(17) Vocalization
(18) Fear (faeces, urine)
(19) Dehydration (skin pinch test/saggy skin)
(20) Discharge from nares/eyes
(21) Wound abnormalities (specify: e.g. inflammation, opened…)
(22) Skin/mucosa colour: normal (N), pale (P), cyanosis (C)
(23) Exploring on top of the platform?
Marking scale
* for all criteria but breathing and skin/mucous membrane colour:
• Absent criterion: 0
• Doubtful or mildly present: 1
• Present: 2
* for type of breathing
• Normal (N): 0
• Moderately laboured: 1
• Laboured (L): 2
• Open‐mouth breathing/noisy breathing: 4
* for skin/mucosa colour
• Normal (N): 0
• Pale (P): 2
• Cyanosis (C): 4
Total mark out of 50

Mice were assessed first from a distance, then following opening of the cage, before finally being handled.

### Mouse Grimace Scale

Mice were placed on an elevated platform (dimensions, 9.5 cm × 6.2 cm, height 36.4 cm) and acclimated for 5 min before photographs were taken over a period of 15–20 min. This confined mice to a small area, without restraint, and in an open position to ensure good quality photographs. Photographs were selected for quality *a posteriori* to obtain three left and three right profile shots and four frontal shots. Photographs were cropped to include the head only and assigned a random number using a home‐written Bash script. All photographs were scored by two independent observers blinded to mouse identity using the criteria described in the initial MGS paper and manual (Langford *et al*. 2010). The five criteria (orbital tightening, nose bulge, cheek bulge, ear position and whisker changes) were assessed, and a score of 0 was given if the criterion was absent, 1 if moderately present and 2 if obviously present.

### Analgesia

All mice scoring greater than 3/50 on the welfare scoring system were defined as ‘in pain’ and received an extra dose of 0.012 mg of buprenorphine s.c. [average dose, 0.6 mg kg^−1^ (SD, 0.1)]. Mice with a score <3 were defined as ‘not in pain’, and a random set of these mice also received buprenorphine. Pain was reassessed after 30 min by the same observer using both systems.

### Infarct size

Infarct sizes were measured using *in vivo* MRI as previously described (Schneider *et al*. 2006). Briefly, 8 weeks after surgery, cine‐MRI data were acquired using a 9.4 T MR system (Agilent Technologies, Santa Clara, CA, USA). Infarct size was measured with ImageJ (version 1.44o; National Institutes of Health, Bethesda, MD, USA) as a percentage of the entire left ventricle.

### Statistical analysis

All data were analysed using GraphPad Prism version 5.0 (GraphPad Software, Inc., La Jolla, CA, USA). Type II regression analysis using Deming's method was performed to correlate interobserver variability of the MGS. A Wilcoxon matched pairs signed rank test was used for comparison before and after analgesia. A Kruskal–Wallis test with Dunn's *post hoc* test was used to assess the effect of the procedure and infarct size on MGS score. All results were considered significant when *P* < 0.05. The MGS data were used in two ways: (i) taking the mean of two observers; or (ii) the mode of the observers’ scores to represent a typical ‘bedside’ assessment. Unless otherwise stated, data are expressed as means ± SD.

## Results

### Interobserver variability

**Figure 1:**
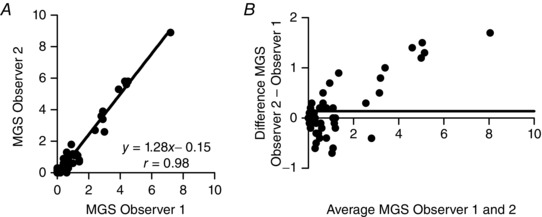
**Interobserver comparison for pain assessment using the Mouse Grimace Scale (MGS) at 24 h postsurgery (*n* = 60 mice before administration of a second dose of buprenorphine)** *A* shows very good correlation between observers (Deming's regression). However, Bland–Altman analysis (*B*) indicates systematic bias, for one observer *versus* the other, particularly at the upper range of MGS scores. Continuous horizontal line represents the mean difference (*n* = 60).

There was an excellent correlation between the two observers for MGS measurement (*r* = 0.98; Fig. 1 *A*). However, the slope differed from unity, as illustrated by Bland–Altman analysis, which showed a systematic bias for higher MGS scores in one of the observers (Fig. 1 *B*).

### Comparison of MGS with traditional scoring systems

**Figure 2:**
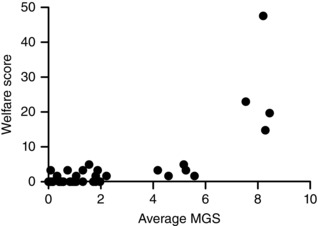
**Comparison between standard welfare scoring and average Mouse Grimace Scale (MGS) for all surgically prepared mice (*n* = 46)**

There was poor correlation between MGS score and body weight loss at 24 h after surgery (*r* = 0.48, data not shown). However, there appeared to be a link between the traditional welfare scoring system and average MGS score in all surgical mice (Fig. 2), especially in mice showing obvious levels of pain and scoring high in both the welfare system and the MGS. An important consideration is whether the MGS is more sensitive for identifying pain in mice that would otherwise be considered normal using the welfare scoring system (i.e. that score <3/50). Notably, 24 mice that scored zero on the welfare assessment registered a score on the MGS. One way to determine whether this represents previously unrecognized ‘real’ pain is to look for an improvement in response to analgesia.

### Effect of analgesia on pain score

**Figure 3:**
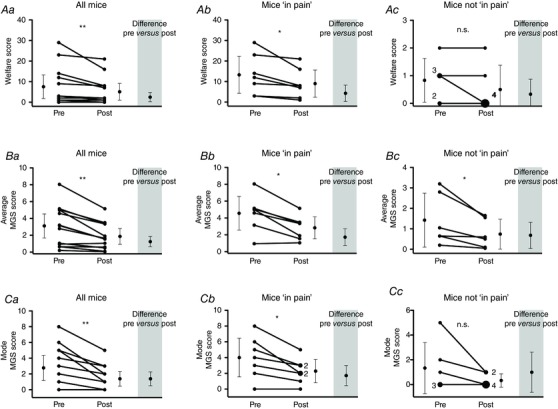
**Effect of buprenorphine analgesia on pain assessed at 24 h postsurgery** Assessments were made before and 30 min after s.c. injection using the following three methods: a welfare scoring sheet (*Aa*, *Ab* and *Ac*); the average MGS score of two blinded observers (*Ba*, *Bb* and *Bc*); and the mode of the MGS score to represent a single ‘on‐the‐spot’ examination (*Ca*, *Cb* and *Cc*). All scoring systems identified a significant improvement following administration of analgesia when all mice (*n* = 13) were analysed together (*Aa*, *Ba* and *Ca*) or when only mice readily identifiable as ‘in pain’ were included (*n* = 7), i.e. welfare score ≥3 (*Ab*, *Bb* and *Cb*). However, only the average MGS method detected an improvement in mice that were otherwise not considered in pain (*n* = 6), i.e. welfare score <3 (*Ac*, *Bc* and *Cc*). All data were analysed using a Wilcoxon matched pairs signed rank test. ‘n.s.’ denotes non‐significant; *P < 0.05 and **P < 0.01. For all graphs, the single points represent single mice, unless otherwise stated. The bars are means ± 95% confidence intervals.

Mice were compared before and 30 min after administration of buprenorphine, which was given regardless of clinical need. Analysis of all mice showed a significant improvement in scores regardless of the assessment system. The average welfare score decreased by 33% (Fig. 3 *Aa*) and the average MGS score by 40% (*P* = 0.003; Fig. 3 *Ba*). Subgroup analysis showed that this was driven mainly by improvements in mice that were deemed to be ‘in pain’ prior to analgesia (Fig. 3 *Ab* and *Bb*). Mice ‘not in pain’ did not show any benefit from analgesia when scored using the welfare system (Fig. 3 *Ac*), but the same mice improved significantly when assessed by MGS (average decrease of 48%, *P* = 0.04; Fig. 3 *Bc*). This suggests that the low‐level pain detected by this type of MGS is real, because it is treatable by analgesia. More typically, the MGS would be scored by a single observer in real time as an on‐the‐spot pain assessment tool, and to simulate these conditions we re‐analysed our data using the mode of the observers’ scores. In these conditions, an improvement was observed only in mice that were previously identified as ‘in pain’ (Fig. 3 *Ca* and *Cb*), and the technique was not sensitive enough to identify an improvement in the ‘not in pain’ group (Fig. 3 *Cc*).

### Effect of infarct size on pain score

**Figure 4:**
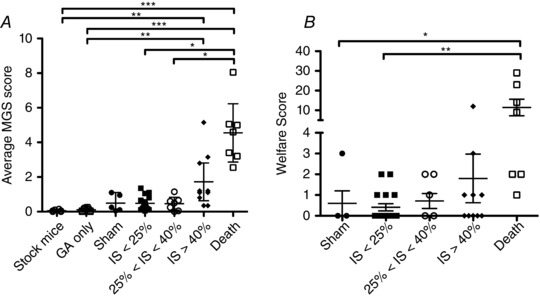
**Contribution of myocardial infarct size (IS) to pain assessment by traditional welfare score (*A*) and average Mouse Grimace Scale (MGS) at 24 h postsurgery (*B*)** ‘Death’ includes all mice that died or were killed before infarct size could be measured. By definition, infarct size is likely to be large in these mice. ‘GA’ denotes mice that received isoflurane general anesthesia but not surgery. Data were analysed using a Kruskal–Wallis test, with Dunn's *post hoc* test; *P < 0.05, **P < 0.01 and ***P < 0.001. Stock mice, *n* = 6; GA only, *n* = 8; sham, *n* = 5; IS <25%, *n* = 17; 25% < IS < 40%, *n* = 7; IS > 40%, *n* = 10; and death, *n* = 7. The bars are means ± SEM.

To determine whether myocardial infarction contributed to pain experienced at 24 h postsurgery, we stratified mice according to MRI‐derived infarct size. In addition, we included a ‘death’ group (*n* = 7), which included mice that survived surgery but which subsequently died before infarct size could be measured at the end of the study. This group consisted of two mice that were killed 24 h after surgery (continued poor welfare scoring after buprenorphine dosing triggered our humane end‐point), one that died of cardiac rupture at day 4, and the others that died due to heart failure at days 3, 22, 49 and 52 after surgery. All stock control mice (no surgery) had very low MGS scores (mean 0.06 ± 0.06), indicating a low level of false positives. Exposure to general anaesthesia increased variability, but did not significantly alter MGS (GA group mean, 0.11 ± 0.10). In sham mice (which underwent thoracotomy only) and in mice with infarct size (IS) <40%, the pain levels measured by the MGS were very similar (0.5 ± 0.5 in sham, 0.5 ± 0.4 for IS <25%, and 0.5 ± 0.4 for IS 25–40%). However, in mice with very large infarcts (IS >40%), the average MGS scores were higher (1.7 ± 1.5) and even higher in those mice that would go on to die before the end of the 8 week protocol (4.6 ± 1.8; Fig. 4 *A*). When the same mice were assessed using the welfare score criteria, all but one of the infarcted mice with IS <40% (*n* = 28) had a welfare score <3 and would therefore be considered as ‘not in pain’ according to traditional criteria (Fig. 4 *B*).

## Discussion

The main finding of this study is that detailed *post hoc* analysis of the Mouse Grimace Scale is a sensitive tool for the assessment of complex postsurgical pain. Using this system, we demonstrated that a significant number of mice are in low‐level pain 24 h after thoracotomy and would benefit from further analgesia. These mice could not be identified using traditional welfare scoring methods, nor by MGS when applied as an on‐the‐spot pain assessment tool. Most of this pain is associated with thoracotomy, with pain from myocardial infarction only contributing when infarct size is particularly large (>40%).

For pain assessment, we scored the intensity of five facial expressions as described by Langford *et al*. (2010) from 10 high‐quality photographs for each mouse. This was performed blind by two independent observers, who received no formal training and scored purely on the basis of the information contained in the MGS manual (provided on request by Dr Jeffrey Mogil, McGill University, Montreal, QC, Canada). The correlation of scores between observers was good, but with a slight systematic bias. This suggests that the facial changes induced by pain are easily detected even by novice observers, but that interpretation of intensity is more subjective. Ideally, MGS scoring should therefore be performed by the same observer if subtle changes in pain levels are to be assessed serially in the same animal.

It is important to note that we used the mean score of both observers for most of our analyses. In this way, we obtained non‐integer scores on what is normally a whole number scale. This proved to be particularly sensitive, because we observed a significant proportion of mice scoring on the MGS but not on the welfare‐based system. This raises the possibility that a large subset of mice were experiencing low‐level pain, which is missed when using standard assessment methods. The fact that the MGS score improved in these mice following administration of an extra dose of buprenorphine strongly suggests that this pain is real and not simply an artefact or caused by noise in our analysis system. Although this decrease is moderate in absolute value (0.7 points on a scale of 10), it represents a reduction by half of the average MGS score following administration of buprenorphine and is likely to be physiologically relevant. Therefore, MGS proved to be very sensitive in detecting pain in a mouse population, but the technique originally described remains labour intensive, with scoring of 10 individual photographs, hence unpractical at the ‘bedside’. Although MGS has been suggested as a quick and easy method for assessing postsurgical pain in individual mice (e.g. Leach *et al*. 2012), this has not been extensively studied and to simulate this, we re‐analysed our data to report the mode score of the observers (i.e. a whole number scale). It is notable that in this format the MGS could clearly identify improvements in animals that had clinical manifestations of pain, but was not sensitive enough to detect improvements in the low‐level pain group. This suggests that on‐the‐spot MGS observations are not any better than standard welfare scoring sheets for identifying mice with postoperative pain. In contrast, blinded, multi‐observer, *post hoc* MGS analysis provides greater sensitivity and represents a useful tool for assessing analgesic protocols in a population of mice. This study has led to a change in our practice, and we now give a second dose of postoperative buprenorphine to all mice as standard.

Previous studies have compared MGS with automated behavioural analysis and found that it was better at detecting the effect of postoperative analgesia (Leach *et al*. 2012). One explanation is that MGS is thought to reflect an integrated response to pain, with an associated affective component (Langford *et al*. 2010; Leach *et al*. 2012). The other consideration is that mice are prey animals and will therefore adapt to pain to prevent appearing vulnerable to predators. For this reason, behavioural changes are mainly observed with acute surgical pain (hours) but are less pronounced in longer‐lasting pain (days; Roughan & Flecknell, 2001; Mogil *et al*. 2010; Urban *et al*. 2011; Matsumiya *et al*. 2012). This represents the main limitation of behavioural‐based methods. However, Langford *et al*. (2010) showed that this can also be a limitation for MGS, where noxious stimuli of moderate duration (10 min to 4 h) gave high MGS score, whereas pain lasting hours to days was undetected.

### Effect of infarct size on pain score

Pain score levels were the same in sham mice (that underwent thoracotomy only) and in mice with small to moderate infarct sizes. This implies that the main source of pain 24 h following the MI procedure is from the thoracotomy rather than pain directly related to ligation of the coronary artery. Death during the postsurgical follow‐up period was predominantly due to heart failure or cardiac rupture, which are highly dependent on infarct size (Lygate, 2006). It is therefore a reasonable assumption that infarct size was particularly large in these mice. These mice, together with mice that had large infarcts (>40%) had very high MGS values, but differences were less pronounced on the welfare scoring system. As the clinical symptoms of heart failure (e.g. changes in mobility, grooming and respiration) are more likely to be reflected in the welfare scoring system, this suggests that very large infarcts may be associated with pain, which persists at 24 h.

### Choice of analgesia protocol

Buprenorphine is the most frequently used analgesic to treat postoperative pain in research laboratories in the UK (Hawkins, 2002) owing to its high efficacy, mild side‐effects and relatively long duration of action (Karas, 2006; Matsumiya *et al*. 2012). The dose of buprenorphine is extremely variable between laboratories, and the ∼0.6 mg kg^−1^ dose used in our study at 24 h is relatively high (Wolfensohn & Lloyd, 2003; Matsumiya *et al*. 2012). Although higher levels can be tolerated (Gades *et al*. 2000), it seems unlikely that underdosing explains why MGS scores did not reduce to control levels. It seems more likely that the efficacy of buprenorphine was submaximal at the time of re‐assessment 30 min postdosing, despite the fact that pharmacokinetic studies show a very fast onset of action following s.c. administration (Cowan *et al*. 1977).

### Limitations

To assess the ability of MGS to screen for individual animals as being in pain, the mode of multiple scores was used to represent a single on‐the‐spot ‘bedside’ examination. Although this is only a proxy for real‐time MGS assessment, it was considered the best way to obtain an unbiased single MGS score, because a true ‘bedside’ scoring system would have been unduly influenced by general mouse behaviour and condition.

Although a statistically significant improvement was observed in the average MGS of mice ‘not in pain’ following administration of buprenorphine, the number of animals assessed was low, and replication on a larger population would allow better generalization of the results.

### Conclusion

In conclusion, *post hoc* analysis of MGS by multiple blinded observers was used to assess pain following thoracotomy and myocardial infarction. Our findings suggest that a significant subset of mice were in low‐level pain at 24 h postsurgery that was undetectable by standard welfare scoring methods or on‐the‐spot MGS assessment. These findings are likely to be applicable to all other surgeries requiring thoracotomy, e.g. cardiac ischaemia–reperfusion or transverse aortic constriction, and we now routinely provide a second dose of analgesic to all mice. Furthermore, this experimental approach represents a template for the assessment of analgesic protocol efficacy in other complex postsurgical models, thereby contributing to the advancement of the ‘3Rs’.

## Additional information

### Competing interests

None declared.

### Author contributions

All experiments were performed at the Wellcome Trust Centre for Human Genetics, University of Oxford, UK. K.M.E.F. and C.A.L. designed the study. K.M.E.F and D.J.McA. performed the experiments. K.M.E.F and C.A.L. analysed and interpreted the data. J.E.S. provided supervision and technical and material support. K.M.E.F. and C.A.L. wrote the draft of the manuscript. All authors critically reviewed the manuscript and approved the final version for publication.

### Funding

This work was supported by the British Heart Foundation (grant numbers RG/13/8/30266 and FS/07/065 to K.M.E.F.), and the authors acknowledge support from Wellcome Trust Core Award, Grant 090532/Z/09/Z. J.E.S. is a British Heart Foundation Senior Basic Science Research Fellow (FS/11/50/29038).

## References

[eph1572-bib-0001] Alderman EL (1974). Analgesics in the acute phase of myocardial infarction. JAMA 229, 1646–1648. 4408159

[eph1572-bib-0002] Carbone L (2011). Pain in laboratory animals: the ethical and regulatory imperatives. Plos One 6, e21578. 21915253 10.1371/journal.pone.0021578 PMC3168441

[eph1572-bib-0003] Carbone L (2012). Pain management standards in the eighth edition of the *Guide for the Care and Use of Laboratory Animals* . J Am Assoc Lab Anim Sci 51, 322–328. 22776189 PMC3358980

[eph1572-bib-0004] Cowan A , Lewis JW & Macfarlane IR (1977). Agonist and antagonist properties of buprenorphine, a new antinociceptive agent. Br J Pharmacol 60, 537–545. 409448 10.1111/j.1476-5381.1977.tb07532.x PMC1667394

[eph1572-bib-0005] Gades NM , Danneman PJ , Wixson SK & Tolley EA (2000). The magnitude and duration of the analgesic effect of morphine, butorphanol, and buprenorphine in rats and mice. Contemp Top Lab Anim Sci 39, 8–13. 11487232

[eph1572-bib-0006] Gerner P (2008). Postthoracotomy pain management problems. Anesthesiol Clin 26, 355–367, vii. 18456219 10.1016/j.anclin.2008.01.007 PMC2453516

[eph1572-bib-0007] Hawkins P (2002). Recognizing and assessing pain, suffering and distress in laboratory animals: a survey of current practice in the UK with recommendations. Lab Anim 36, 378. 12396281 10.1258/002367702320389044

[eph1572-bib-0008] Institute for Laboratory Animal Research (2011). Guide for the Care and Use of Laboratory Animals, 8th edn. National Academies Press, Washington, DC, USA.

[eph1572-bib-0009] Karas AZ (2006). Barriers to assessment and treatment of pain in laboratory animals. Lab Anim (NY) 35, 38–45. 16807565 10.1038/laban0706-38

[eph1572-bib-0010] Kilkenny C , Browne WJ , Cuthill IC , Emerson M & Altman DG (2010). Improving bioscience research reporting: the ARRIVE guidelines for reporting animal research. PLoS Biol 8, e1000412. 20613859 10.1371/journal.pbio.1000412 PMC2893951

[eph1572-bib-0011] Langford DJ , Bailey AL , Chanda ML , Clarke SE , Drummond TE , Echols S , Glick S , Ingrao J , Klassen‐Ross T , Lacroix‐Fralish ML , Matsumiya L , Sorge RE , Sotocinal SG , Tabaka JM , Wong D , van den Maagdenberg AMJM , Ferrari MD , Craig KD & Mogil JS (2010). Coding of facial expressions of pain in the laboratory mouse. Nat Methods 7, 447–449. 20453868 10.1038/nmeth.1455

[eph1572-bib-0012] Leach MC , Klaus K , Miller AL , Scotto Di Perrotolo M , Sotocinal SG & Flecknell PA (2012). The assessment of post‐vasectomy pain in mice using behaviour and the Mouse Grimace Scale. PLoS ONE 7, e35656. 22558191 10.1371/journal.pone.0035656 PMC3338444

[eph1572-bib-0013] Lygate C (2006). Surgical models of hypertrophy and heart failure: Myocardial infarction and transverse aortic constriction. Drug Dis Today Dis Models 3, 283–290.

[eph1572-bib-0014] Lygate C & Neubauer S (2006). Surgically induced chronic heart failure In A Handbook of Mouse Models of Cardiovascular Disease, ed. Xu Q, pp. 335–350. John Wiley & Sons, Chichester , UK.

[eph1572-bib-0015] Matsumiya LC , Sorge RE , Sotocinal SG , Tabaka JM , Wieskopf JS , Zaloum A , King OD & Mogil JS (2012). Using the Mouse Grimace Scale to reevaluate the efficacy of postoperative analgesics in laboratory mice. J Am Assoc Lab Anim Sci 51, 42–49. 22330867 PMC3276965

[eph1572-bib-0016] Mogil JS , Graham AC , Ritchie J , Hughes SF , Austin J‐S , Schorscher‐Petcu A , Langford DJ & Bennett GJ (2010). Hypolocomotion, asymmetrically directed behaviors (licking, lifting, flinching, and shaking) and dynamic weight bearing (gait) changes are not measures of neuropathic pain in mice. Mol Pain 6, 34. 20529328 10.1186/1744-8069-6-34 PMC2893131

[eph1572-bib-0017] Morton DB (1999). Humane endpoints in animal experimentation for biomedical research: ethical, legal and practical aspects In Humane Endpoints in Animal Experimentation for Biomedical Research, ed. Hendriksen CFM & Morton DB, pp. 5–12. The Royal Society Medical Press, London, UK.

[eph1572-bib-0018] Patten RD , Aronovitz MJ , Deras‐Mejia L , Pandian NG , Hanak GG , Smith JJ , Mendelsohn ME & Konstam MA (1998). Ventricular remodeling in a mouse model of myocardial infarction. Am J Physiol Heart Circ Physiol 274, H1812–H1820. 10.1152/ajpheart.1998.274.5.H1812 9612394

[eph1572-bib-0019] Roughan JV & Flecknell PA (2001). Behavioural effects of laparotomy and analgesic effects of ketoprofen and carprofen in rats. Pain 90, 65–74. 11166971 10.1016/s0304-3959(00)00387-0

[eph1572-bib-0020] Salto‐Tellez M , Yung Lim S , El‐Oakley RM , Tang TP , ALmsherqi ZAM & Lim SK (2004). Myocardial infarction in the C57BL/6 J mouse: a quantifiable and highly reproducible experimental model. Cardiovasc Pathol 13, 91–97. 15033158 10.1016/S1054-8807(03)00129-7

[eph1572-bib-0021] Schneider JE , Wiesmann F , Lygate CA & Neubauer S (2006). How to perform an accurate assessment of cardiac function in mice using high‐resolution magnetic resonance imaging. J Cardiovasc Magn Reson 8, 693–701. 16891228 10.1080/10976640600723664

[eph1572-bib-0022] Urban R , Scherrer G , Goulding EH , Tecott LH & Basbaum AI (2011). Behavioral indices of ongoing pain are largely unchanged in male mice with tissue or nerve injury‐induced mechanical hypersensitivity. Pain 152, 990–1000. 21256675 10.1016/j.pain.2010.12.003 PMC3079194

[eph1572-bib-0023] Wallis J , Lygate CA , Fischer A , ten Hove M , Schneider JE , Sebag‐Montefiore L , Dawson D , Hulbert K , Zhang W , Zhang MH , Watkins H , Clarke K & Neubauer S (2005). Supranormal myocardial creatine and phosphocreatine concentrations lead to cardiac hypertrophy and heart failure: insights from creatine transporter‐overexpressing transgenic mice. Circulation 112, 3131–3139. 16286605 10.1161/CIRCULATIONAHA.105.572990

[eph1572-bib-0024] Wolfensohn S & Lloyd M (2003). Pain, stress and humane end points In Handbook of Laboratory Animal Management and Welfare, 3rd edn, ed. Wolfensohn S & Lloyd M, pp. 59–73. Blackwell Publishing Ltd, Oxford, UK.

